# Characterization of phenylalanine hydroxylase gene variants and analysis of genotype–phenotype correlation in patients with phenylalanine hydroxylase deficiency from Fujian Province, Southeastern China

**DOI:** 10.1007/s11033-022-07579-8

**Published:** 2022-09-14

**Authors:** Jinfu Zhou, Yinglin Zeng, Xiaolong Qiu, Qingying Lin, Weifeng Chen, Jinying Luo, Liangpu Xu

**Affiliations:** 1 Medical Genetic Diagnosis and Therapy Center, Fujian Maternity and Child Health Hospital College of Clinical Medicine for Obstetrics & Gynecology and Pediatrics, Fujian Medical University, Fuzhou, 350001 Fujian Province China; 2 Obstetrics and Gynecology Department, Fujian Maternity and Child Health Hospital College of Clinical Medicine for Obstetrics & Gynecology and Pediatrics, Fujian Medical University, Fuzhou, 350001 Fujian Province China

**Keywords:** Phenylalanine hydroxylase deficiency, Variant spectrum, Genotype–phenotype correlation, Prediction, Southeastern China

## Abstract

**Background:**

Phenylalanine hydroxylase deficiency (PAHD) is the most prevalent inherited disorder of amino acid metabolism in China. Its complex phenotype includes many variants and genotypes among different populations.

**Methods and results:**

In this study, we analyzed the phenylalanine hydroxylase gene (*PAH*) variants in a cohort of 93 PAHD patients from Fujian Province. We also assessed genotype and phenotype correlation in patients with PAHD. A total of 44 different pathogenic variants were identified, including five novel variants. The three most prevalent variants among all patents were c.158G > A, p.(Arg53His) (18.03%), c.721C > T, p.(Arg241Cys) (14.75%), and c.728G > A, p.(Arg243Gln) (7.65%). The frequency of the c.158G > A, p.(Arg53His) variant was highest in patients with mild hyperphenylalaninemia, whereas the frequency of the c.1197A > T, p.(Val399 =) and c.331C > T, p.(Arg111Ter) variants was highest in patients with classic phenylketonuria. The most abundant genotypes observed in PAHD patients were c.[158G > A];[728G > A], c.[158G > A];[442-1G > A], and c.[158G > A];[721C > T]. Comparing allelic phenotype to genotypic phenotype values yielded fairly accurate predictions of phenotype, with an overall consistency rate was 85.71% for PAHD patients.

**Conclusions:**

Our study identified a *PAH* variant spectrum in PAHD patients from Fujian Province, Southeastern China. Quantitative correlation analysis between genotype and phenotype severity is helpful for genetic counseling and management.

## Introduction

Phenylalanine hydroxylase deficiency (PAHD, MIM#261,600) is an autosomal recessive metabolic disorder caused by variants in the gene encoding the enzyme phenylalanine hydroxylase (PAH; EC 1.14.16.1). PAHD is the most prevalent inborn genetic defect of the amino acid metabolism seen in China, with an average incidence of approximately 1:12,000 [1]. However, the incidence rate varies significantly across different regions, ranging from 1:30,000 to 1:2,000 [2–5]. PAH catalyzes the hydroxylation of L-phenylalanine (L-Phe), forming L-tyrosine using tetrahydrobiopterin (BH4) as a cofactor. *PAH* variants lead to a loss in enzyme activity and an increase in serum concentrations of phenylalanine (Phe). Abnormal accumulation of Phe in serum can damage the peripheral and central nervous systems, resulting in mental retardation, seizures, and cerebral palsy to varying degrees if left untreated [6]. The mechanisms of neurotoxicity caused by elevated phenylalanine levels include impairing the synthesis of brain catecholamines, cholesterol, and proteins; extensively disturbing neutral amino acid transportation to the brain; inhibiting pyruvate kinase; dysregulating calcium levels; and inducing mitochondrial dysfunction, oxidative stress, and inflammation [6–8]. Based on the severity of the metabolic phenotype, PAHD is classified into three types: classic phenylketonuria (cPKU), mild PKU (mPKU), and mild hyperphenylalaninemia (MHP). Therefore, early diagnosis and treatment of PKU are essential in avoiding permanent damage.

The human *PAH* gene comprises 13 exons and 12 introns, which are located on chromosome 12q23.2. To date, more than 1,200 different variants have been reported in the *PAH* gene of patients with PAHD and have been registered in the Phenylalanine Hydroxylase Gene Locus-Specific database (PAHdb; http://www.pahdb.mcgill.ca). The detection of *PAH* variants and the analysis of the correlation between the genotype and clinical phenotype are extremely valuable for genetic counseling, the selection of the most suitable therapeutic options, and prognosis prediction [9–13]. Although several genotype-based methods for phenotype prediction have been applied for this disease, further clarification on their predictive value is required [14–17]. In addition, the great regional and ethnic heterogeneity of *PAH* variants necessitates the establishment of a spectrum of *PAH* variants in a population-specific manner. Certain studies have reported the characteristics of *PAH* gene variants in different populations, including the Chinese population [2, 3, 17–19]. However, the spectra of *PAH* variants show certain variations among the populations in different provinces of China [2, 3, 18]. This necessitates the establishment of a *PAH* gene variant spectrum among the PAHD patients in Fujian Province, Southeastern China.

In this study, we analyzed the *PAH* variants in a cohort of 93 PAHD patients from Fujian Province using next-generation sequencing (NGS) and Sanger sequencing with the aim of characterizing the distribution of *PAH* variants in this region. Additionally, we analyzed the genotype and phenotype correlation in patients with PAHD.

## Materials and methods

### Study subjects

A total of 93 children (56 males and 37 females) diagnosed at the Medical Genetic Diagnosis and Therapy Center of Fujian Province Maternal and Child Health Hospital between January 2016 and September 2021 were included in this study. All patients and their parents were from the Chinese Han population.

All patients were identified through a neonatal hyperphenylalaninemia screening program. We applied tandem mass spectrometry to measure plasma phenylalanine concentrations from dried blood samples before treatment was started. All patients had plasma Phe levels of > 120 μmol/L, and Phe:Tyr ratios of > 2. Additionally, a urinary pterin analysis and dihydropteridine reductase activity assay were performed on dried blood spot samples to exclude patients with tetrahydrobiopterin reductase deficiency. The metabolic phenotypes of the patients were classified according to their plasma Phe concentrations before treatment, and the maximum pretreatment values were applied in this study. The patients were diagnosed with cPKU (Phe ≥ 1,200 μmol/L), mPKU (Phe: 360–1200 μmol/L), or MHP (Phe: 120–360 μmol/L) [20].

### Genotype analysis

Genomic DNA was isolated from the peripheral blood using the QIAamp DNA Mini Kit (Qiagen, Germany), following the manufacturer’s instructions. NGS was used to detect the *PAH* gene variants using Biosan (Zhejiang, China). The basic edition panel of inherited metabolic diseases was used to detect 94 genes, including *PAH*, *PTS*, *GCH1*, *QDPR*, and *PCBD1*. Briefly, the target region sequences were enriched by multiple probes, and then, the capture products were purified. Thereafter, library construction, sequencing, and data analysis were carried out. Then, the detected variants were verified using Sanger sequencing. To determine sequence variability, the genes of the respective parents were screened for the variables detected in their offspring.

The obtained sequences were compared with the wild-type transcript of human *PAH* (NM_0002777.3) to identify potential variants. Variant nomenclature was adherent to the guidelines and recommendations of the Human Genome Variation Society (http://varnomen.hgvs.org/). Gene variants were classified according to the ACMG guidelines (https://clinicalgenome.org/).

To exclude polymorphic sites in the population, all detected gene variants were tested against the 1000 Genomes Project, dbSNP, and ExAC databases. In addition, variants were screened against the ClinVar database (http://www.ncbi.nlm.nih.gov/clinvar/), Human Gene variant database (http://www.hgmd.cf.ac.uk/ac/index.php), and PAHdb to identify potential unreported variants (novel variants). Two online tools, Provean (http://provean.jcvi.org/) and PolyPhen-2 (http://genetics.bwh.harvard.edu/pph2/dokuwiki/start), were used to predict the pathogenicity of *PAH* missense variants, and variantTaster (https://www.varianttaster.org) was used to predict the pathogenic effects of variants at splicing sites.

### De novo variants pedigrees

Paternity testing was subsequently conducted to determine the biological nature of the relationship between the patient and their parents, with the aim of confirming that the de novo variants were not inherited from parents*.*

### Genotype–phenotype prediction

Two different algorithms were used to analyze the correlation between genotype and phenotype. Based on the in vitro residual activity associated with the *PAH* variants [14], the gene variants were predicted to cause any of the three phenotypes: cPKU (21.1% ± 7.0%), mPKU (40.2% ± 7.6%), and MHP ( 52.1% ± 8.5%). In addition, the allelic phenotype values (APV) of the *PAH* variants were queried in the BIOPKU database (http://www.biopku.org). Phenotypes were predicted using genotypic phenotype values (GPVs) [15], which were equal to the higher APV alleles: (i) GPV = 0–2.7 for cPKU, (ii) GPV = 2.8–6.6 for mPKU, and (iii) GPV = 6.7–10 for MHP [15]. We were unable to predict the phenotype of one variant that did not have an APV score in the BIOPKU database. Finally, we defined these alleles as null alleles, such as frameshift, splice-site, and nonsense variants.

### Statistical analyses

All categorical data were expressed as proportions. Comparisons between the distribution frequencies of *PAH* variants in the different subtypes of the disorder were performed using the χ^2^ test. All statistical analyses were implemented using the statistical software SPSS 17.0. *P* value < 0.05 was considered to be statistically significant.

## Results

### Variant spectrum of the *PAH* gene

**Fig. 1:**
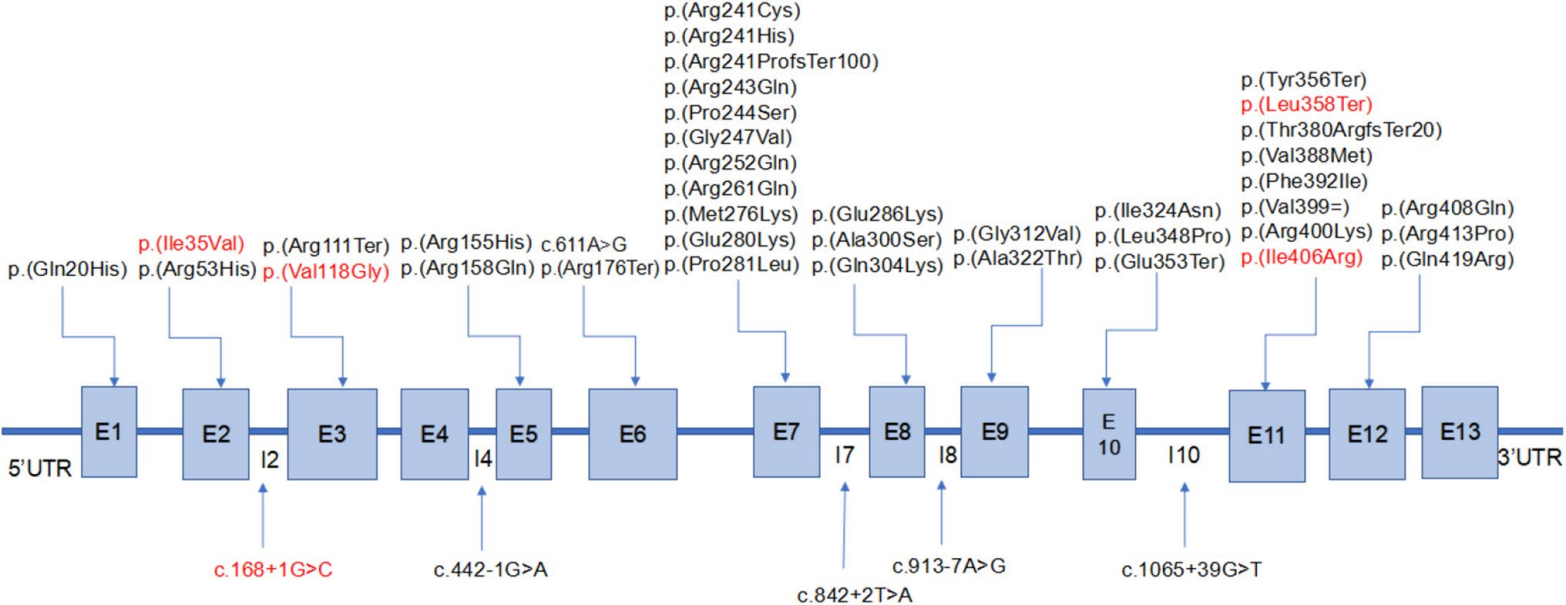
Forty-four variants were identified in PAHD patients from Fujian Province. Novel variants identified in this study are depicted in red. *E* exon; *I* intron

A total of 93 unrelated patients with PAHD were investigated, including 14 cPKU, 20 mPKU, and 59 MHP patients. Further, 183 variant alleles were detected from the 186 alleles of PAHD patients. A total of 44 different pathogenic variants were identified, including 35 (79.54%) missense variants, 6 (13.64%) nonsense variants, 7 (15.91%) splice-site variants, and 2 (4.54%) frameshift deletions (Table 1). At the amino acid sequence level, the nine most prevalent variants represented 51.36% of the total, and were c.158G > A, p.(Arg53His) (18.03%), c.721C > T, p.(Arg241Cys) (14.75%), c.728G > A, p.(Arg243Gln) (7.65%), c.1174 T > A, p.(Phe392Ile) (6.01%), c.1197A > T, p.(Val399 =) (6.01%), c.331C > T, p.(Arg111Ter) (4.92%), c.1223G > A, p.(Arg408Gln) (4.92%), c.611A > G, p.(?) (4.37%), and c.442-1G > A, p.(?) (2.73%) (Table 1). Variants were unevenly distributed in the *PAH* gene. The variants with the highest frequencies of variants were located in exons 7 (30.05%), 2 (18.57%), 11 (17.48%), 12 (7.65%), 6 (5.46%), and 9 (4.37%). No variants were detected in exons 4 and 13 (Fig. 1). The distribution of variants in different regions of China is summarized in Table 2.

**Table 1:** PAH mutations in PAH-deficient patients from Fujian province

cDNA aberration	Protein effect or trivial name	Gene region	Type	Protein domain	No. of alleles	RF (relative frequency) (%)
c.158G > A	p.(Arg53His)	Exon 2	Missense	Regulatory	33	18.03
c.721C > T	p.(Arg241Cys)	Exon 7	Missense	Catalytic	27	14.75
c.728G > A	p.(Arg243Gln)	Exon 7	Missense	Catalytic	14	7.65
c.1174 T > A	p.(Phe392Ile)	Exon 11	Missense	Catalytic	11	6.01
c.1197A > T	p.(Val399 =)	Exon 11	Splice/Silence	Catalytic	11	6.01
c.1223G > A	p.(Arg408Gln)	Exon 12	Missense	Catalytic	9	4.92
c.331C > T	p.(Arg111Ter)	Exon 3	Nonsense	Regulatory	9	4.92
c.611A > G	p.(?)	Exon 6	Splice	Catalytic	8	4.37
c.913-7A > G	p.(?)	Intron 8	Splice	–	5	2.73
c.442-1G > A	p.(?)	Intron 4	Splice	–	5	2.73
c.1162G > A	p.(Val388Met)	Exon 11	Missense	Catalytic	3	1.64
c.1238G > C	p.(Arg413Pro)	Exon 12	Missense	Catalytic	3	1.64
c.722del	p.(Arg241ProfsTer100)	Exon 7	Frame shift deletion	Catalytic	3	1.64
c.856G > A	p.(Glu286Lys)	Exon 8	Missense	Catalytic	3	1.64
c.1199G > A	p.(Arg400Lys)	Exon 11	Missense	Catalytic	2	1.09
c.1217 T > G	p.(Ile406Arg)	Exon 11	Missense	Catalytic	2	1.09
c.1256A > G	p.(Gln419Arg)	Exon 12	Missense	Catalytic	2	1.09
c.464G > A	p.(Arg155His)	Exon 5	Missense	Catalytic	2	1.09
c.526C > T	p.(Arg176Ter)	Exon 6	Nonsense	Catalytic	2	1.09
c.755G > A	p.(Arg252Gln)	Exon 7	Missense	Catalytic	2	1.09
c.782G > A	p.(Arg261Gln)	Exon 7	Missense	Catalytic	2	1.09
c.842C > T	p.(Pro281Leu)	Exon 7	Missense	Catalytic	2	1.09
c.935G > T	p.(Gly312Val)	Exon 9	Missense	Catalytic	2	1.09
c.103A > G	p.(Ile35Val)	Exon 2	Missense	Regulatory	1	0.055
c.1043 T > C	p.(Leu348Pro)	Exon 10	Missense	Catalytic	1	0.055
c.1057G > T	p.(Glu353Ter)	Exon 10	Nonsense	Catalytic	1	0.055
c.1065 + 39G > T	p.(?)	Intron 10	Splice	–	1	0.055
c.1068C > A	p.(Tyr356Ter)	Exon 11	Nonsense	Catalytic	1	0.055
c.1073 T > G	p.(Leu358Ter)	Exon 11	Nonsense	Catalytic	1	0.055
c.1138del	p.(Thr380ArgfsTer20)	Exon 11	Frame shift deletion	Catalytic	1	0.055
c.168 + 1G > C	p.(?)	Intron 2	Splice	–	1	0.055
c.353 T > G	p.(Val118Gly)	Exon 3	Nonsense	Regulatory	1	0.055
c.473G > A	p.(Arg158Gln)	Exon 5	Missense	Catalytic	1	0.055
c.60G > C	p.(Gln20His)	Exon 1	Missense	Regulatory	1	0.055
c.722G > A	p.(Arg241His)	Exon 7	Missense	Catalytic	1	0.055
c.730C > T	p.(Pro244Ser)	Exon 7	Missense	Catalytic	1	0.055
c.740G > T	p.(Gly247Val)	Exon 7	Missense	Catalytic	1	0.055
c.827 T > A	p.(Met276Lys)	Exon 7	Missense	Catalytic	1	0.055
c.838G > A	p.(Glu280Lys)	Exon 7	Missense	Catalytic	1	0.055
c.842 + 2 T > A	p.(?)	Intron 7	Splice	–	1	0.055
c.898G > T	p.(Ala300Ser)	Exon 8	Missense	Catalytic	1	0.055
c.910C > A	p.(Gln304Lys)	Exon 8	Missense	Catalytic	1	0.055
c.964G > A	p.(Ala322Thr)	Exon 9	Missense	Catalytic	1	0.055
c.971 T > A	p.(Ile324Asn)	Exon 10	Missense	Catalytic	1	0.055

**Table 2:** Comparisons of the PAH gene variant frequencies between PAHD from Fujian and other regions in China

cDNA aberration	Protein effect or trivial name	Fujian (%)	Eastern China [3] (%)	North China [5] (%)	South China [2] (%)	Northwest China [24] (%)	Central China [18] (%)
c.158G > A	p.(Arg53His)	18.03	4.34	4.7	15.2		5.37
c.721C > T	p.(Arg241Cys)	14.75	13.27	4.6	13.8		7.38
c.728G > A	p.(Arg243Gln)	7.65	22.19	17.7	17.2	14.00	16.11
c.1174 T > A	p.(Phe392Ile)	6.01	1.79				6.04
c.1197A > T	p.(Val399 =)	6.01	0.77	6.4	5.2		
c.1223G > A	p.(Arg408Gln)	4.92	2.81				
c.331C > T	p.(Arg111Ter)	4.92	5.36	4.4		2.95	
c.611A > G	p.(?)	4.37	8.93	8.3	20.7	5.58	10.07
c.913-7A > G	p.(?)	2.73	0.77				
c.442-1G > A	p.(?)	2.73	5.61	3.4	5.2	4.32	
c.1238G > C	p.(Arg413Pro)	1.64		4.6	6.9	4.74	
c.722del	p.(Arg241ProfsTer100)	1.64			1.7		
c.464G > A	p.(Arg155His)	1.09			3.4		
c.1068C > A	p.(Tyr356Ter)	0.055	3.06	4.7	1.7	4.95	4.70

### Genotype analysis of PAHD patients

The frequency of variants that occurred in more than 1% of the patients were compared among the three subgroups of PAHD patients’ six variants: c.158G > A, p.(Arg53His) (*P* = 0.000), c.721C > T, p.(Arg241Cys) (*P* = 0.001), c.1174 T > A, p.(Phe392Ile) (*P* = 0.034), c.1197A > T, p.(Val399 =) (*P* = 0.036), c.331C > T, p.(Arg111Ter) (*P* = 0.03), and c.1199G > A, p.(Arg400Lys) (*P* = 0.027). The variants exhibited significantly different frequencies across the three patient phenotypes. c.1174 T > A, p.(Phe392Ile) and c.1199G > A, p.(Arg400Lys) were only detected in patients with MHP and mPKU, respectively. c.1223G > A, p.(Arg408Gln) was detected only in patients with MHP and mPKU. Finally, the c.158G > A, p.(Arg53His) variant was more frequent in patients with MHP, while c.1197A > T, p.(Val399 =) and c.331C > T, p.(Arg111Ter) variants were more frequent in patients with cPKU compared to the other subgroups (Table 3).

**Table 3:** PAH allele distribution in PAHD patients from Fujian province

cDNA Aberration	Protein effect or trivial name	Classical PKU (n = 27)		Moderate PKU (n = 40)		MHP (n = 116)		*P* value
		No. of alleles	RF (%)	No. of alleles	RF (%)	No. of alleles	RF (%)	
c.158G > A	p.(Arg53His)	0	0.00	1	2.50	32	27.58	0.000^#^
c.721C > T	p.(Arg241Cys)	1	3.70	13	32.5	13	11.21	0.001^#^
c.728G > A	p.(Arg243Gln)	1	3.70	4	10.00	9	7.76	0.635
c.1174 T > A	p.(Phe392Ile)	0	0.00	0	0.00	11	9.48	0.034^#^
c.1197A > T	p.(Val399 =)	5	18.51	2	5.00	4	3.45	0.036^#^
c.1223G > A	p.(Arg408Gln)	1	3.70	4	10.00	4	3.45	0.243
c.331C > T	p.(Arg111Ter)	4	14.81	2	5.00	3	2.59	0.03^#^
c.611A > G	p.(?)	3	11.11	2	5.00	3	2.59	0.145
c.442-1G > A	p.(?)	0	0.00	0	0.00	5	4.31	0.098
c.913-7A > G	p.(?)	0	0.00	2	5.00	3	2.59	0.463
c.856G > A	p.(Glu286Lys)	0	0.00	2	5.00	1	0.86	0.158
c.722del	p.(Arg241ProfsTer100)	1	3.70	0	0.00	2	1.72	0.500
c.1256A > G	p.(Gln419Arg)	0	0.00	0	0.00	2	1.72	0.558
c.1199G > A	p.(Arg400Lys)	0	0.00	2	5.00	0	0.00	0.027^#^
c.1217 T > G	p.(Ile406Arg)	1	3.70	0	0.00	1	0.86	0.333
c.464G > A	p.(Arg155His)	0	0.00	0	0.00	2	1.72	0.558
c.526C > T	p.(Arg176Ter)	1	0.00	0	0.00	2	1.72	0.558
c.755G > A	p.(Arg252Gln)	1	3.70	0	0.00	1	0.86	0.333
c.782G > A	p.(Arg261Gln)	0	0.00	0	0.00	2	1.72	0.558
c.842C > T	p.(Pro281Leu)	0	0.00	1	2.50	1	0.86	0.538
c.935G > T	p.(Gly312Val)	0	0.00	0	0.00	2	1.72	0.580

*PKU* phenylketonuria, *cPKU* classic phenylketonuria, *mPKU* mild phenylketonuria, *MHP* mild hyperphenylalaninemia, *RF* relative frequency

^#^Significant difference among the three PAHD phenotypes

Out of the 93 patients, 96.77% (90/93) carried biallelic variants and were either homozygous (n = 3) or compound heterozygous (n = 87). Monoallelic variants were detected in three patients, one of which presented the cPKU phenotype, whereas the other two presented the MHP phenotype.

At the amino acid level, 63 distinct combinations were detected in 90 patients (Table 4), including the two homoallelic genotypes, c.[1223G > A];[1223G > A] (1.11%) and c.[721C > T];[721C > T] (2.22%). The most abundant genotypes observed were c.[158G > A];[728G > A] (6.67%), c.[158G > A];[442-1G > A] (4.44%), and c.[158G > A];[721C > T] (3.33%), and were only carried by MHP patients.

**Table 4:** Genotype–phenotype correlations in PAHD patients

Genotype	Protein effect	Residual activity [%]*	Mean activity [%]**	Predicted phenotype based on activity***	GPV	Predicted Phenotype based on GPV	Observed phenotype			RF[%]
							cPKU	mPKU	MHP	
c.[1068C > A];[721C > T]	p.[Tyr356Ter];[Arg241Cys]	null/57	28.5	cPKU-mPKU	5.5	mPKU		1		1.11
c.[1138del];[158G > A]	p.[Thr380ArgfsTer20];[Arg53His]	null/79	39.5	mPKU	9.5	MHP			1	1.11
c.[1162G > A];[158G > A]	p.[Val388Met];[Arg53His]	28/79	53.5	MHP	9.5	MHP			1	1.11
c.[1162G > A];[721C > T]	p.[Val388Met];[Arg241Cys]	28/57	42.5	mPKU	5.5	mPKU		1	1	2.22
c.[1174 T > A];[331C > T]	p.[Phe392Ile];[Arg111Ter]	98/null	49	MHP	8	MHP			1	1.11
c.[1174 T > A];[721C > T]	p.[Phe392Ile];[Arg241Cys]	98/57	77.5	MHP	8	MHP			2	2.22
c.[1174 T > A];[842C > T]	p.[Phe392Ile];[Pro281Leu]	98/2	50	MHP	8	MHP			1	1.11
c.[1174 T > A];[422-1G > A]	p.[Phe392Ile];[?]	98/null	49	MHP	8.0	MHP			1	1.11
c.[1197A > T];[158G > A]	p.[Val399 =];[Arg53His]	null/79	39.5	mPKU	9.5	MHP			2	2.22
c.[1197A > T];[782G > A]	p.[Val399 =];[Arg261Gln]	null/44	22	cPKU	1.6	cPKU			1	1.11
c.[1197A > T];[331C > T]	p.[Val399 =];[Arg111Ter]	null/null	0	cPKU	0.7	cPKU	1	1		2.22
c.[1197A > T];[728G > A]	p.[Val399 =];[Arg243Gln]	null/14	14	cPKU	0.7	cPKU		1		1.11
c.[1197A > T];[1217 T > G]	p.[Val399 =];[Ile406Arg]	null/n.d	n.d	n.d	n.d	n.d	1			1.11
c.[1197A > T];[611A > G]	p.[Val399 =];[?]	null/null	0	cPKU	0.7	cPKU	1			1.11
c.[1199G > A];[842C > T]	p.[Arg400Lys];[Pro281Leu]	n.d./0	n.d	n.d	0	cPKU		1		1.11
c.[1199G > A];[721C > T]	p.[Arg400Lys];[Arg241Cys]	n.d./57	n.d	n.d	5.5	mPKU		1		1.11
c.[1223G > A];[1174 T > A]	p.[Arg408Gln];[Phe392Ile]	41/98	69.5	MHP	8.0	MHP			2	2.22
c.[1223G > A];[721C > T]	p.[Arg408Gln];[Arg241Cys]	41/57	49	MHP	5.5	mPKU			2	2.22
c.[1223G > A];[722G > A]	p.[Arg408Gln];[Arg241His]	41/23	32	mPKU	5.2	mPKU		1		1.11
c.[1223G > A];[1223G > A]	p.[Arg408Gln];[Arg408Gln]	41/41	41	mPKU	5.2	mPKU		1		1.11
c.[1223G > A];[331C > T]	p.[Arg408Gln];[Arg111Ter]	41/null	20.5	cPKU	5.2	mPKU		1		1.11
c.[1238G > C];[721C > T]	p.[Arg413Pro];[Arg241Cys]	11/57	34	mPKU	5.2	mPKU	1	1		2.22
c.[1238G > C];[331C > T]	p.[Arg413Pro];[Arg111Ter]	11/null	5.5	cPKU	0	cPKU	1			1.11
c.[1256A > G];[971 T > A]	p.[Gln419Arg];[Ile324Asn]	72/n.d	n.d	n.d	9.7	MHP			1	1.11
c.[103A > G];[158G > A]	p.[Ile35Val];[Arg53His]	n.d./79	n.d	n.d	n.d	n.d			1	1.11
c.[158G > A];[442-1G > A]	p.[Arg53His];[?]	79/null	39.5	mPKU	9.5	MHP			4	4.44
c.[158G > A];[1073 T > G]	p.[Arg53His];[Leu358Ter]	79/null	39.5	mPKU	9.5	MHP			1	1.11
c.[158G > A];[1217 T > G]	p.[Arg53His];[Ile406Arg]	79/n.d	n.d	n.d	9.5	MHP			1	1.11
c.[158G > A];[331C > T]	p.[Arg53His];[Arg111Ter]	79/null	39.5	mPKU	9.5	MHP			2	2.22
c.[158G > A];[60G > C]	p.[Arg53His];[Gln20His]	79/n.d	n.d	n.d	9.5	MHP			1	1.11
c.[158G > A];[722del]	p.[Arg53His];[Arg241ProfsTer100]	79/null	39.5	mPKU	9.5	MHP			2	2.22
c.[158G > A];[728G > A]	p.[Arg53His];[Arg243Gln]	79/14	46.5	MHP	9.5	MHP			6	6.66
c.[168 + 1G > C];[1174 T > A]	p.?;[Phe392Ile]	null/98	49	MHP	8.0	MHP			1	1.11
c.[473G > A];[464G > A]	p.[Arg158Gln];[Arg155His]	10/44	27	cPKU	10	MHP			1	1.11
c.[526C > T];[1197A > T]	p.[Arg176Ter];[Val399 =]	null/null	0	cPKU	0.7	cPKU	2			2.22
c.[611A > G];[158G > A]	p.[?];[Arg53His]	null/79	39.5	mPKU	9.5	MHP			2	2.22
c.[611A > G];[331C > T]	p.[?];[Arg111Ter]	null/null	0	cPKU	0	cPKU	1			1.11
c.[611A > G];[721C > T]	p.[?];[Arg241Cys]	null/57	28.5	cPKU-mPKU	5.5	mPKU		2		2.22
c.[611A > G];[1043 T > C]	p.[?];[Leu348Pro]	null/25	12.5	cPKU	0	cPKU	1			1.11
c.[721C > T];[158G > A]	p.[Arg241Cys];[Arg53His]	57/79	68	MHP	9.5	MHP			3	3.33
c.[721C > T];[353 T > G]	p.[Arg241Cys];[Val118Gly]	57/n.d	n.d	n.d	n.d	n.d			1	1.11
c.[721C > T];[721C > T]	p.[Arg241Cys];[Arg241Cys]	57/57	57	MHP	5.5	mPKU			2	2.22
c.[721C > T];[728G > A]	p.[Arg241Cys];[Arg243Gln]	57/14	35.5	mPKU	5.5	mPKU		3		3.33
c.[728G > A];[1174 T > A]	p.[Arg243Gln];[Phe392Ile]	14/98	56	MHP	8.0	MHP			2	2.22
c.[728G > A];[1256A > G]	p.[Arg243Gln];[Gln419Arg]	14/71	42.5	mPKU	9.7	MHP			1	1.11
c.[728G > A];[910C > A]	p.[Arg243Gln];[Gln304Lys]	14/n.d	n.d	n.d	n.d	n.d	1			1.11
c.[740G > T];[331C > T]	p.[Gly247Val];[Arg111Ter]	4/null	2	cPKU	0	cPKU	1			1.11
c.[755G > A];[722del]	p.[Arg252Gln];[Arg241ProfsTer100]	15/null	7.5	cPKU	0	cPKU	1			1.11
c.[782G > A];[158G > A]	p.[Arg261Gln];[Arg53His]	44/79	61.5	MHP	9.5	MHP			1	1.11
c.[827 T > A];[158G > A]	p.[Met276Lys];[Arg53His]	35/79	57	MHP	9.5	MHP			1	1.11
c.[842 + 2 T > A];[1223G > A]	p.?;[Arg408Gln]	null/41	20.5	cPKU	5.2	mPKU		1		1.11
c.[856G > A];[158G > A]	p.[Glu286Lys];[Arg53His]	1/79	40	mPKU	9.5	MHP			1	1.11
c.[856G > A];[721C > T]	p.[Glu286Lys];[Arg241Cys]	1/57	29	cPKU-mPKU	5.2	mPKU		2		2.22
c.[898G > T];[464G > A]	p.[Ala300Ser];[Arg155His]	65/44	54.5	MHP	9.7	MHP			1	1.11
c.[913-7A > G];[721C > T]	p.[?];[Arg241Cys]	null/57	28.5	cPKU-mPKU	5.5	mPKU		2		2.22
c.[913-7A > G];[730C > T]	p.[?];[Pro244Ser]	null/n.d./null	n.d	n.d	n.d	n.d		1		1.11
c.[1065 + 39G > T];[1223G > A]	p.[?];[Arg408Gln]	null/41	20.5	cPKU	n.d	n.d	1			1.11
c.[913-7A > G];[1174 T > A]	p.[?];[Phe392Ile]	null/98	49	MHP	8.0	MHP			1	1.11
c.[913-7A > G];[611A > G]	p.[?];[?]	null/null	0	cPKU	0	cPKU			1	1.11
c.[935G > T];[158G > A]	p.[Gly312Val];[Arg53His]	7/79	43	mPKU	9.5	MHP			1	1.11
c.[935G > T];[838G > A]	p.[Gly312Val];[Glu280Lys]	7/11	9	cPKU	0	cPKU			1	1.11
c.[964G > A];[755G > A]	p.[Ala322Thr];[Arg252Gln]	n.d./15	n.d	n.d	0	cPKU			1	1.11

*n.d.* not determined, *PKU* phenylketonuria, *cPKU* classic phenylketonuria, *mPKU* mild phenylketonuria, *MHP* mild hyperphenylalaninemia, *RF* relative frequency, *GPV* genotypic phenotype value

*PAH in vitro residual activity for each mutant protein was assessed from data compiled in the BIOPKUdb [http://www.biopku.org/home/biopku.asp]

**Mean activity is defined as the average sum of activities of both two alleles, and expressed as the percentage of the wild-type enzyme

***The expected phenotypes were predicted by the mean of the two allelic activities. The activity cut-off was the following: cPKU, 21.1% ± 7.0%; mPKU, 40.2% ± 7.6%; MHP, 52.1% ± 8.5%. cPKU-mPKU means the activity was between cPKU and mPKU

### Prediction of genotype–phenotype correlations in PAH-deficient patients

A total of 84 patients met the requirements for phenotype prediction based on the APV/GPV system analysis. As shown in Table 4, this analysis accurately predicted 56.25% (9/16) of cPKU, 77.27% (17/22) of mPKU, and 100% (46/46) of MHP patients. Notably, the prediction coincidence rate of PAHD patients was as high as 85.71% (72/84). Based on the PAH residual activity in vitro, the phenotypic prediction of PAHD patients was consistent with the observed phenotype in 65.82% (52/79) of the PAHD patients, consisting of 55.55% (10/18), 42.42% (14/33), and 100% (28/28) of cPKU, mPKU, and MHP patients, respectively. Finally, the coincidence rate of phenotypic prediction between GPV and residual activity analysis was 75% (35/50) (Table 4).

### Pedigrees of patients with de novo variants

By screening the corresponding loci containing the variant in the parents, we found one patient carrying de novo variant. Subsequently, paternity testing confirmed the biological relationship between the patient and parents. Thus, the de novo variant was further confirmed.

### Novel sequence variants

Five novel variants that were not recorded in the BIOPKU database were detected in this study, comprising three missense variants (c.103A > G, p.(Ile35Val), c.1217 T > G, p.(Ile406Arg), and c.353 T > G, p.(Val118Gly)), one nonsense variant (c.1073 T > G, p.(Leu358Ter)), and one splicing variant (c.168 + 1G > C, p.(?)). The variant c.1217 T > G, p.(Ile406Arg) was observed only in cPKU patients, whereas c.103A > G, p.(Ile35Val), c.353 T > G, p.(Val118Gly), c.1073 T > G, p.(Leu358Ter), and cc.168 + 1G > C, p.(?) were observed only in MHP patients. The deleterious effects of these variants were predicted in silico and are listed in Table 5.

**Table 5:** The silico prediction analysis of the 5 novel variants of the PAH gene detected in this study

NO	Mutation type	cDNA Aberration	Protein effect or trivial name	PolyPhen-2	Provean	Mutation Taster
1	Missense	c.103A > G	p.(Ile35Val)	Benign	Neutral	Polymorphism
2		c.1217 T > G	p.(Ile406Arg)	Probably damaging	Deleterious	Disease causing
3		c.353 T > G	p.(Val118Gly)	Probably damaging	Deleterious	Disease causing
4	Nonsense	c.1073 T > G	p.(Leu358Ter)		Deleterious	Disease causing
5	Splice	c.168 + 1G > C	p.(?)			Disease causing

## Discussion

In this study, 93 patients with PAHD that had been identified via national newborn screening in the past 5 years were included. The variant detection rate was 98.39% (183/186). The high detection rate of gene variants is consistent with previous studies confirming the universality of *PAH* gene variants. Variants in six exons (7, 2, 11, 12, 6, and 9) accounted for 84.57% of the total variants, which is consistent with previous reports in other regions of China and Asia [17, 19, 21–23]. A total of 44 distinct variants were detected, nearly half of which were observed only once, indicating a high degree of genetic heterogeneity among the PAHD population in Fujian Province. Nine variants accounted for 51.36% of the total variants among the PAHD patients, the most common of which were c.158G > A, p.(Arg53His) (18.03%) and c.721C > T, p.(Arg241Cys) (14.75%). However, previous studies reported that c.728G > A, p.(Arg243Gln) was the most prevalent variant in North and Central Chinese populations, accounting for 17.7% and 16.11% respectively [5, 18]. In addition, the predominant variant was c.611A > G, p.(?)(20.7%) in the population of Hainan Province [2]. This inconsistency may be related to the high proportion of MHP patients included in this study and the existence of specific alleles among different regions.

The results of this study show that six variant sites presented frequencies that were significantly different among the three phenotypes of patients. Variants c.158G > A, p.(Arg53His) and c.1174 T > A, p.(Phe392Ile) had relatively high frequencies among MHP patients (*P* = 0.001 and 0.014, respectively), whereas c.721C > T, p.(Arg241Cys) and c.1223G > A, p.(Arg408Gln) had relatively high frequencies in mPKU patients. In addition, variant c.158G > A, p.(Arg53His) was relatively more frequent among MHP patients, in line with the results of previous large-sample studies [3, 24]. In vitro studies have reported that the residual activity of the c.158G > A, p.(Arg53His)-type PAH enzyme was equivalent to 79% of that of the wild-type [14, 25]. Whether the variant c.158G > A, p.(Arg53His) is polymorphic remains controversial. The findings of a previous study suggested that patients with PAHD carrying c.158G > A, p.(Arg53His) did not require frequent Phe monitoring, unlike those with PKU [26]. In line with these results, certain previous studies have regarded variant c.158G > A, p.(Arg53His) as being “likely benign” [3, 4, 27]. Finally, the proportion of patients with MHP in Fujian province was significantly higher than that reported by the BIOPKU database and previous reports from other regions in China. This may be attributed to the higher frequency of the variant c.158G > A, p.(Arg53His) in this region. Finally, variant c.1174 T > A, p.Phe392Ile was only observed in patients with MHP, in line with the results of previous studies [3, 24].

Previous studies showed that the detection rate of variant p.(Arg241Cys) in mPKU and MHP patients from southern China was higher than that in other areas [3, 28, 29]. Interestingly, variant c.721C > T, p.(Arg241Cys) was more frequent in patients with mPKU (32.50%) and MHP (11.21%) compared to that in the cPKU (3.70%) group, suggesting that our study may represent regional and demographic differences to a certain degree. Although Arg241 is localized close to the cofactor binding region, this amino acid is not directly involved in the interaction with the cofactor [28]. Hence, variant c.721C > T, p.(Arg241Cys) causes relatively mild structural changes.

Most of the patients (63/90) included in our study presented a unique genotype, showing that the genotypes in Fujian Province are highly heterogeneous. In addition, 93.54% (87/93) of the patients were compound heterozygotes, a slightly higher proportion that was reported by the BIOPKU database (76%). The most prevalent genotype was c.[158G > A];[728G > A] (6.67%), followed by c.[158G > A];[442-1G > A] (4.44%), and c.[158G > A];[721C > T] (3.33%), which is inconsistent with an earlier finding [30]. This may be related to the high proportion of patients with MHP in this study. The high proportion of patients with MHP may also represent regional and ethnic heterogeneity. Interestingly, two c.721C > T, p.(Arg241Cys) homozygous patients showed a MHP phenotype, which was also found in previous studies [3, 21]. In addition, a total of 12 compound heterozygous variants were detected in the 13 patients with cPKU. At present, prenatal diagnosis is increasingly used to prevent the recurrence of PKU in families [31–33]. Therefore, these results can provide preliminary and valuable data for the prenatal diagnosis and prevention of cPKU.

Previous studies have attempted to uncover a quantitative correlation between the genotype and phenotype in PAHD patients using a series of different algorithms [15, 16, 34–36]. APV and GPV have been identified as systems with high sensitivity and specificity for phenotypic prediction [4, 15]. In this study, the APV/GPV-based prediction system adequately predicted the actual phenotype, as the overall consistency rate was 85.71% for PAHD, and 100% MHP. The APV value of variant c.158G > A, p.(Arg53His) was 9.5, and the phenotype was predicted to be MHP patients. In this study, we found that variant c.158G > A, p.(Arg53His) was closely associated with MHP. These findings strongly support the close correlation between the genotypes and phenotypes underlying PAHD using the APV/GPV-based prediction system. By contrast, the prediction system based on the average value of the residual activity of the two alleles in vitro did not perform well, with a prediction accuracy rate of only 65.82% for PAHD and 42.42% for mPKU. Genotypic and phenotypic inconsistencies existed mainly in patients that had compound heterozygous variants. The mechanism underlying these discrepancies requires further clarification. The co-expression of different *PAH* variants may lead to a residual activity that differs from the predicted activity owing to intermolecular interactions [37]. However, the accuracy of these two prediction systems in predicting PKU needs to be further improved. Therefore, further studies should optimize these systems to accurately predict phenotypes.

Certain studies show that large-scale deletion/duplication variants were related to the pathogenesis of PAHD. In a large cohort of 475 PKU families in Northwest China, 74 cases without two known pathogenic variants were analyzed for large-scale deletion/duplication variants using multiplex ligation-dependent probe amplification (MLPA) method, and 7 large-scale deletion/duplication variants were found in 25 patients (25/475, 5.26%). In another Chinese population study, large-scale deletion/duplication variants were detected in 48 of 808 PAHD patients, accounting for 5.94%. Out of 293 patients with hyperphenylalaninemia in Russia, 10 patients with one PAH gene variant had gross deletions revealed. In the PAHD patients from Italy, the detection rate of large-scale deletion/duplication variants was as low as 1.3% (13/759). Interestingly, there was only one *PAH* gene variant that was detected in three patients in this study. These patients had normal pterin profiles and dihydropteridine reductase activity, and no variants were detected in *PTS*, *GCH1*, *QDPR*, and *PCBD1*. Therefore, a large-scale deletion/replication of the variants might be involved in the pathogenesis in these patients. Unfortunately, the patients were not further assessed using MLPA analysis.

Interestingly, there was one case in which the PKU pedigree identified a novel de novo *PAH* gene variant. The patient exhibited a compound heterozygous variant with c.[1223G > A];[722G > A]. However, the variant c.722G > A was not found in the corresponding locus in either of the parents. After confirming a biological relationship between the patient and the parents through the paternity testing, we considered this to be a true de novo variant. Thus, the identification of de novo variants may enable accurate and appropriate genetic counseling to be provided to the affected families.

In conclusion, we constructed a *PAH* gene variant spectrum of the PAHD patients of Fujian Province and identified novel variants that broaden the *PAH* gene variant spectrum. Exploring and clarifying the differences in the frequencies of different *PAH* gene variants among patients with different sub-phenotypes may elucidate the correlation between compound heterozygosity and phenotype. In addition, we demonstrated that genotype–phenotype prediction using the APV/GPV system resulted in a higher prediction accuracy than when the results of residual enzyme activity in vitro were used, illustrating that APV/GPV prediction could be a suitable tool for genetic counseling of families with PAHD patients.
